# Nanobiosensors: A Potential Tool to Decipher the Nexus Between SARS-CoV-2 Infection and Gut Dysbiosis

**DOI:** 10.3390/s26020616

**Published:** 2026-01-16

**Authors:** Atul Kumar Tiwari, Munesh Kumar Gupta, Siddhartha Kumar Mishra, Ramovatar Meena, Fernando Patolsky, Roger J. Narayan

**Affiliations:** 1 Department of Chemistry, Indian Institute of Technology (BHU), Varanasi 221005, India; 2 Department of Microbiology, Institute of Medical Sciences, Banaras Hindu University, Varanasi 221005, India; 3 Department of Biochemistry, University of Lucknow, Lucknow 226007, India; siddharthakm@yahoo.com; 4 School of Environmental Sciences, Jawaharlal Nehru University, New Delhi 110067, India; 5 School of Chemistry, Tel Aviv University, P.O. Box 39040, Tel Aviv 6997801, Israel; 6 Joint Department of Biomedical Engineering, University of North Carolina, Chapel Hill, NC 27695, USA

**Keywords:** SARS-CoV-2, gut microbiome, dysbiosis, nanotechnology, nanobiosensors

## Abstract

The emergence of SARS-CoV-2 posed a great global threat and emphasized the urgent need for diagnostic tools that are rapid, reliable, sensitive and capable of real-time monitoring of SARS-CoV-2 infections. Recent investigations have identified a potential connection between SARS-CoV-2 infection and gut dysbiosis, highlighting the sophisticated interplay between the virus and the host microbiome. This review article discusses the eminence of nanobiosensors, as state-of-the-art tools, to investigate and clarify the connection between SARS-CoV-2 pathogenesis and gut microbiome imbalance. Nanobiosensors are uniquely advantageous owing to their sensitivity, selectivity, specificity, and reliable monitoring capabilities, making them well-suited for identifying both viral particles and microbial markers in biological samples. We explored a range of nanobiosensor platforms and their potential use for concurrently monitoring the gut dysbiosis induced by different pathological conditions. Additionally, we explore how advanced sensing technologies can shed light on the mechanisms driving virus-induced dysbiosis, and the implications for disease progression and patient outcomes. The integration of nanobiosensors with microfluidic devices and artificial intelligence algorithms has also been explored, highlighting the potential of developing point-of-care diagnostic tools that provide comprehensive insights into both viral infection and gut health. Utilizing nanotechnology, scientists and healthcare professionals may gain a more profound insight into the complex interaction dynamics between SARS-CoV-2 infection and the gut microenvironment. This could pave the way for enhanced diagnostic and prognostic approaches, treatment courses, and patient care for COVID-19.

## 1. Introduction

SARS-CoV-2 emerged as an enormous threat to global health and safety. As of September 2025, the virus has affected more than 779 million people and caused over seven million deaths worldwide [1]. SARS-CoV-2 spreads through inhalation or contact with droplets and aerosols expelled by infected individuals during coughing or sneezing. Once the virus reaches the nasal cavity, it penetrates the host epithelial cells via the ACE2 receptor, which is abundantly present on epithelial cells, in both the respiratory and digestive systems [2,3]. Infections usually trigger a swift innate immune response that helps control or eliminate the virus, resulting in mild symptoms. In certain instances, the virus persists in the lower respiratory tract, leading to increased pro-inflammatory reactions and severe consequences, such as acute respiratory distress syndrome, organ failure, and death [4]. SARS-CoV-2 affects not only the respiratory system, but also other organ systems [5]. Approximately 55% of patients show prolonged viral RNA in their feces, even weeks after viral clearance from the respiratory tract [6]. Factors such as abnormal immune responses, comorbidities, and advanced age are linked to COVID-19 severity; however, they do not fully explain severe disease outcomes in all patients. Given the ongoing difficulties with widespread COVID-19 vaccination and treatment, it is crucial to explore new approaches and methods for safeguarding and caring for those affected by the virus [7,8]. The human microbiome plays a crucial role in establishing and maintaining immune balance, and research has shown that disruptions in the microbiota balance, or dysbiosis, are closely linked to various diseases. The intestine and mouth, which house the largest and second largest microbiota populations in the human body, respectively, are pivotal in the development of infectious diseases. Studies have indicated that microbes originating from the oral cavity and lungs can influence the course of various infectious diseases by altering the host mucosal immune response [9,10,11]. The gut microbiota influences the onset and development of viral infections via the gut–lung axis [12,13,14]. Conversely, viral infections can alter the microbiome, resulting in changes in vulnerability and severity of diseases due to dysbiosis [15]. Early research has confirmed a clear link between influenza virus and bacterial co-infection and disease severity [16]. In this context, nanotechnology could be pivotal in swiftly diagnosing, monitoring, and creating effective treatments for COVID-19, especially in terms of how SARS-CoV-2 affects the gut microbiome. For example, breath tests utilizing nanomaterials-based arrays can identify volatile organic compounds (VOCs) that carry microbiota signatures directly affected by SARS-CoV-2 infection, such as VOCs originating from the presence of the bacteria Prevotella, thereby allowing the detection of SARS-CoV-2 for prompt diagnosis and monitoring. Additionally, ingestible sensors may be engineered in future to identify, in-gut, the inflammatory proteins associated with COVID-19 infection.

## 2. The Gut Microbiome and Gut–Lung Nexus

**Figure 1:**
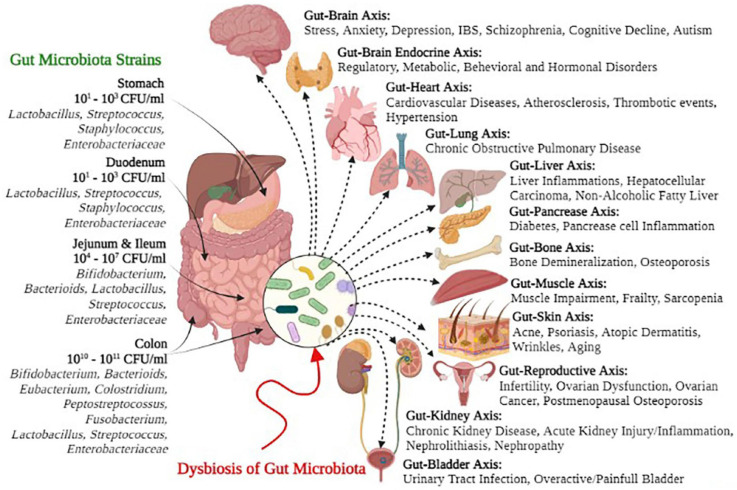
Gut microbial dysbiosis is associated with an imbalance or disruption in the composition and diversity of the gut microbiome. This condition is linked to negative health outcomes (e.g., gastrointestinal disorders, metabolic diseases, and immune dysfunction). Specific gut microbial strains are associated with gut health; their absence or overgrowth is associated with dysbiosis. For example, a decrease in beneficial bacteria (e.g., *Bifidobacterium* and *Lactobacillus*) is associated with inflammatory bowel diseases, while an increase in harmful bacteria (e.g., *E. coli* and *C. difficile*) is associated with intestinal infections and inflammation. Adopted with permission from ref. [18].

The human digestive tract hosts a diverse array of beneficial microbes, collectively known as gut microbiota. It is deemed that the digestive system harbors more than 10^14^ microorganisms, which contain hundred times more DNA than the human genome [17]. The gut microbiome comprises approximately 1000–1500 bacterial species, with each individual hosting approximately 160 unique species. In the gut, the predominant phyla are *Firmicutes* and *Bacteroidetes*, while in the lungs, *Proteobacteria*, *Bacteroidetes*, and *Firmicutes* are the most commonly observed (Figure 1) [14,18]. Generally, the gut microbiota provides numerous benefits to the host, including the suppression of harmful pathogens, maintenance of gut integrity, breakdown of undigested substances, and development of a tolerant barrier in the mucosa and intestinal epithelium. The relationship between the immune system and gut microbiota is intricate; 70–80% of the immune cells of the body are located in the gut [18,19]. While most research has concentrated on defining a healthy baseline community, microbiomes outside the gut are also essential, even though their functions and disease-related variations have not been specifically investigated [20,21]. The oral microbiome has been examined in relation to dental cavities and periodontal disease [22,23,24], while the vaginal microbiome is associated with bacterial vaginosis [25,26] and an increased risk of yeast and viral infections [27]. The skin microbiome is linked to conditions such as atopic dermatitis, acne, and psoriasis, and may play a role in activating the immune system and enhancing resistance to infections [28,29,30,31]. Additionally, the microbiome has been implicated in an increased risk of melanoma [32,33]. Research on the gut microbiome has also demonstrated strong associations with various diseases, including obesity, type-2 diabetes, cirrhosis, and rheumatoid arthritis [32,33]. Mouse models have been utilized to investigate the potential mechanisms linking the gut microbiome to these conditions, as well as to anxiety, depression, and even autism [34]. Maternal immune activation via double-stranded RNA viruses can lead to autism-like behavior and gut barrier dysfunction in offspring, partially reversible using the probiotic *Bacteroides fragilis* [34].

The stability of the immune system is intricately connected to the microbiome, which delivers vital signals, including microbial components and metabolites, which are essential for the proper development and function of the immune system [35]. In humans, factors such as dietary habits, antibiotic use, and stress can disrupt the balance of gut bacteria, leading to a decline in beneficial bacteria and an increase in harmful bacteria [36]. This imbalance, termed dysbiosis, can disturb the equilibrium of tissues and the immune system, and is linked to various inflammatory diseases, both within and beyond the gastrointestinal tract [37]. For example, when communication between the intestine and lungs is compromised, the likelihood of respiratory diseases and infections, including allergies, can increase [38]. The importance of the gut–lung axis is evident in patients with chronic gastrointestinal disorders, such as irritable bowel syndrome (IBS) and inflammatory bowel disease (IBD), who are more prone to respiratory diseases [38,39,40]. The epithelial lining of both the gastrointestinal and respiratory systems encounters a wide variety of microorganisms, with ingested microbes potentially reaching these areas. Furthermore, microbiota present in the gastrointestinal tract can migrate to the lungs through micro-aspiration. The mucosal surfaces of the gut and respiratory tract act as barriers against microbial invasion, whereas normal microbiota aid in pathogen defense by producing bacteriocins [41]. Additionally, commensal gut bacteria, including segmented filamentous bacteria (SFB), *Bifidobacterium* spp., and *Bacteroides* spp., promote antimicrobial peptides, secretory immunoglobulin A (sIgA), and pro-inflammatory cytokines [42]. Non-pathogenic strains of *Salmonella* reduce inflammatory reactions in gastrointestinal epithelial cells by inhibiting the ubiquitination of nuclear factor-κB (NF-κB) inhibitor-α (IκBα). Meanwhile, certain species of *Clostridium* promote anti-inflammatory responses by enhancing regulatory T cell (Treg cell) activity [42,43].

**Figure 2:**
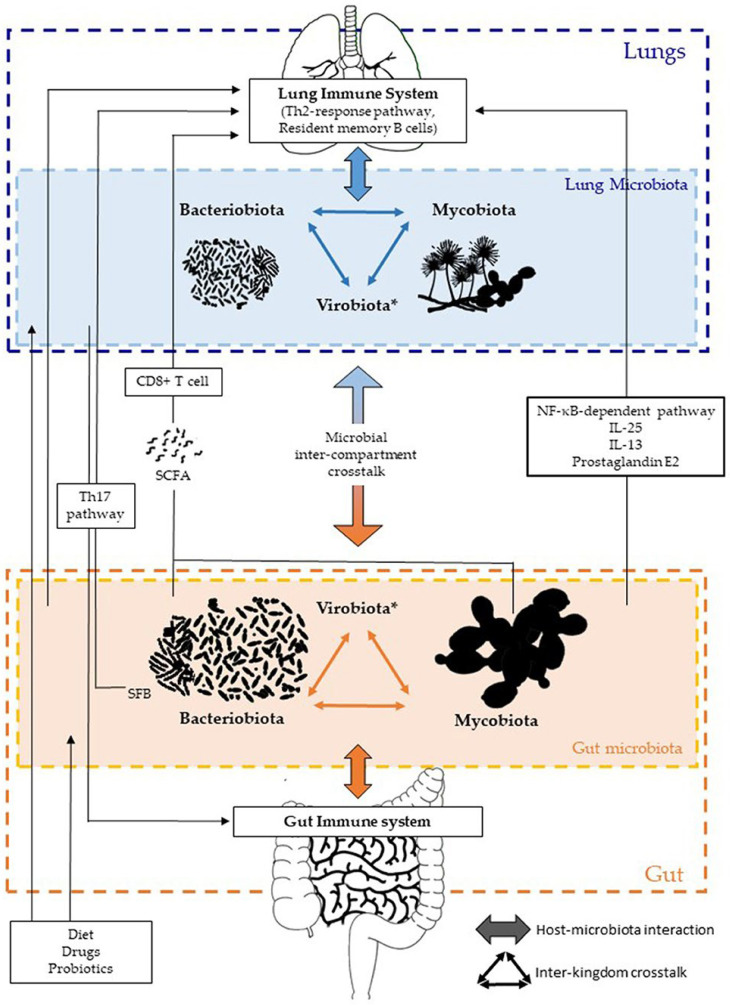
Communication between different kingdoms and compartments within the gut–lung axis involves complex interactions. Bacteriobiota, mycobiota, and virobiota* (gut viral composition) engage each organ through both direct and indirect methods. The commensal microbiota influences the immune systems of both the gut and lungs through a combination of local and widespread interactions involving CD8+ T cells, Th17, IL-25, IL-13, prostaglandin E2, and NF-κB pathways. The lung microbiota contributes to mucosal immunity and immune tolerance by attracting neutrophils, generating pro-inflammatory cytokines through receptor 2 (TLR2), and releasing antimicrobial peptides activated by T helper 17 (Th17) cells. Additionally, lung microbiota affects the gut immune system, although the mechanisms are not fully understood, with disruptions in intestinal microbes associated with Th17 cell mediation following influenza virus infection in the lungs. Factors such as diet, medications, and probiotics can alter the composition of intestinal and lung microbiota. Adopted with permission from ref. [14].

Within the respiratory system, the interaction between *S. pneumoniae* and *H. influenzae* triggers the host’s p38 mitogen-activated protein kinase (MAPK) without relying on toll-like receptors (TLR), which, in turn, enhances proinflammatory responses [44]. Conversely, non-pathogenic *S. pneumoniae* and other bacterial species, along with their components, can alleviate allergic airway diseases by promoting the growth of Treg cells [45,46,47,48]. In individuals who have undergone lung transplantation, alterations in the respiratory tract microbiota can significantly affect lung immunity. Disparities in the levels of *Firmicutes*, *Proteobacteria*, and *Bacteroidetes* are linked to the expression of inflammatory genes in lung leukocytes, whereas an imbalance in *Bacteroides* is associated with the expression of genes related to tissue remodeling [49]. Investigations using cell cultures and animal models have demonstrated that pathogenic species elicit a stronger inflammatory response than commensal microorganisms do. This suggests that various lung microbiota might offer protection against diseases by dampening pro-inflammatory signals from pathogens [50,51]. Although fecal suspension transfer techniques have been employed to explore gut microbiota, these methods have not been adapted for transferring respiratory microbiota between animals, which restricts our understanding of their functions. Recent findings suggest that host epithelial cells, along with other structural and immune cells, receive signals from microorganisms and local cytokine responses. This interaction influences inflammatory reactions and shapes immune responses in distant areas, such as the lungs (Figure 2) [14,52]. While the direct transfer of microorganisms between different sites is not extensively documented, there is evidence of bacterial migration from the gut to the lungs in conditions such as sepsis and acute respiratory distress syndrome, where the integrity of barriers is compromised [53]. Additionally, environmental influences, such as dietary fiber, can induce similar alterations in the microbiota of both the gut and lungs [54]. The precise factors responsible for these effects, whether they stem from changes in microbial metabolites due to dietary shifts, alterations in immune responses, or a combination of both, remain unclear.

## 3. Diet and Gut Microbiome

Research has demonstrated that dietary choices can significantly determine the composition of the gut microbiota [55]. The gut microbiome (GM) predominantly consists of the phylum *Firmicutes* (~64%), which encompasses genera such as *Lactobacillus*, *Eubacterium*, *Bacillus*, *Enterococcus*, *Clostridium*, *Ruminococcus*, *Faecalibacterium*, and *Roseburia*. The next most abundant phylum is *Bacteroidetes* (<23%), which includes genera like *Bacteroides* and *Prevotella*, followed by *Actinobacteria* (<3%) and *Verrucomicrobia* (<2%) [18,56,57]. Nonetheless, the composition of GM can vary widely among individuals, influenced by factors such as age, genetic makeup, birth method, infant feeding practices (breast milk or formula), antibiotic usage, geographic location, and diet [58,59]. Diet is recognized as a pivotal factor impacting GM and is characterized by a complex bidirectional relationship. GM composition can affect nutrient absorption and metabolism, potentially influencing the physiological processes of the host. Furthermore, the composition and function of the GM can be influenced by nutrients, bioactive compounds, certain foods, and dietary habits, leading to either positive or negative effects on human health [60]. For those with asymptomatic COVID-19, mild symptoms, or those in quarantine, it is advisable to follow a nutritious and balanced diet that includes cereals, whole grains, legumes, fruits, and vegetables.

This nutritional strategy is based on the negative correlation between the consumption of dietary fiber and the serum levels of strong inflammatory cytokines, such as interleukin-6 (IL-6), interleukin-18 (IL-18), C-reactive protein, and tumor necrosis factor-alpha (TNFα). Fiber-rich diets are associated with reduced glucose levels and higher plasma adiponectin concentrations [61]. The gut microbiota rapidly adjusts to short- and long-term dietary changes, showing daily variability and the ability to double within an hour [62]. Acetylation of histone deacetylase 3 (HDAC3) in epithelial cells is strongly linked to alterations in the gut microbiome. HDAC3 is involved in the circadian rhythm and influences food consumption by controlling the expression of genes related to metabolism. This interaction influences lipid consumption, contributing to diet-induced obesity [63,64]. The feeding schedule, including timing, duration, frequency, and type, significantly influences the composition, function, and health of the gut microbiota. Kaczmarek et al. discovered that meal timing is associated with the presence of specific bacteria [65]. Similarly, Thaiss Et Al. demonstrated in mouse models that rhythmic food intake increased microbial abundance and caused 15% fluctuations in the commensal microbiota throughout the day [66]. In a 2018 randomized crossover study, Collado Et Al. examined the effects of meal timing on human gut microbiota [67]. Dietary habits exhibit a cyclical seasonal pattern influenced by both availability and routine, with these routines having a more profound impact on the gut microenvironment and compositional complexity than daily fluctuations [68]. The initial three years of life are pivotal in forming the microbial environment, with diet playing a significant role [69]. By the time a child reaches three years of age, a stable, adult-like gut microbiota is established, providing enhanced resistance to opportunistic infections. Interestingly, children possess a more complex microbial diversity than healthy adults [70]. In a cross-sectional study conducted by Hollister Et Al., pre-adolescent children had a more varied diet than adults. While adults’ dietary patterns are often shaped by lifestyle and food availability, children are more likely to try new foods [71,72]. Despite these differences, the amount and quality of nutrients can influence the gut microbiota. Nonetheless, other factors, such as genetics and lifestyle habits, also contribute to the development of the gut microbiota and should not be ignored.

## 4. SARS-CoV-2 Infection and Gut Microbiome

**Figure 3:**
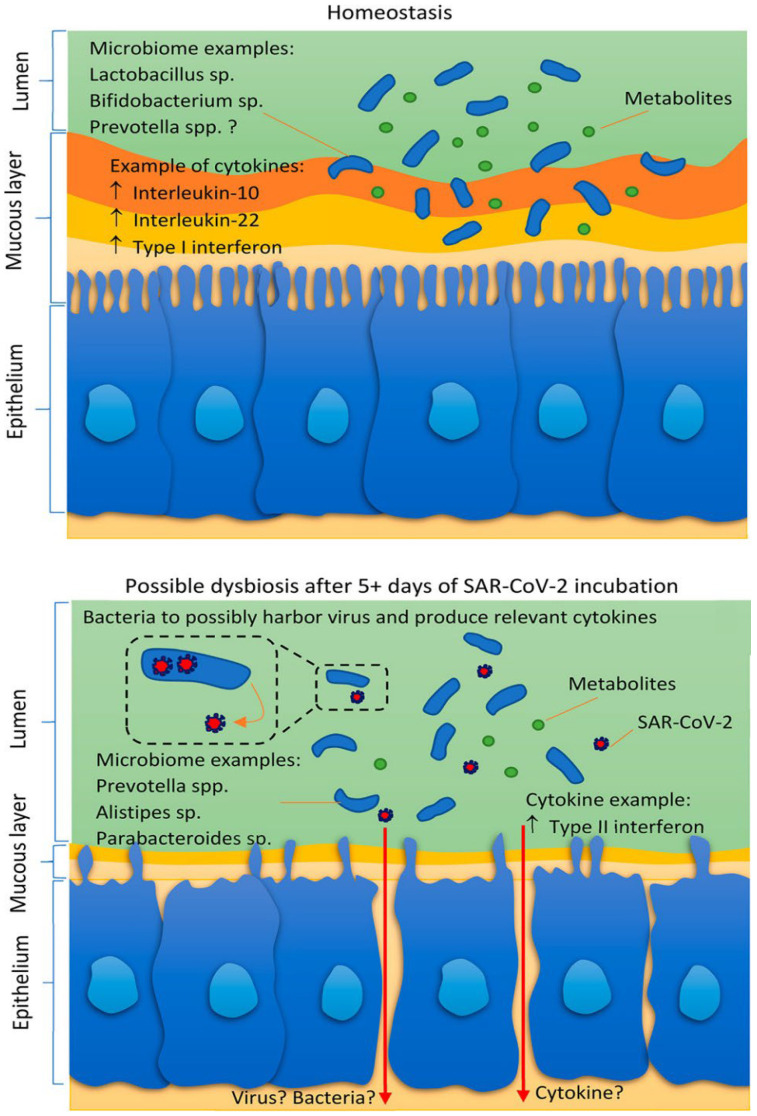
SARS-CoV-2 can upset the equilibrium between host homeostasis and viral infection, potentially causing dysbiosis across various organs. This imbalance may result in modifications to the gut microbiome, changes in the microbial community of the respiratory tract, or even systemic shifts in immune responses, all of which contribute to the intricate pathophysiology of COVID-19. Adopted with permission from ref. [74].

Hence, it is vital to assess the role of the gut in alleviating or intensifying SARS-CoV-2 infection. Viruses can modify the gut environment and commensal microbiota, leading to either amplified or diminished effects [73]. Thus, it is prudent to explore how interactions between the gut and SARS-CoV-2 might influence the severity of infection and clinical outcomes. The integrity of the gut microbiome, which includes the collective genomes of various microorganisms within the human gastrointestinal tract, can be compromised by SARS-CoV-2 infection, leading to gut dysbiosis (Figure 3) [74]. Evidence indicates a connection between gut function and the response of the microbiome to SARS-CoV-2 infection. For example, the incubation period for SARS-CoV-2 generally lasts 5–6 days, whereas for influenza, it is approximately two days. Furthermore, diarrhea can present as an early indicator of SARS-CoV-2 [74,75,76]. Recent findings have proposed that the virus could be transmitted through the fecal-oral pathway [77]. Individuals at the highest risk of experiencing severe symptoms and fatality from SARS-CoV-2 include older adults and those with pre-existing medical conditions, such as diabetes, which is linked to inflammation and other health issues [78]. Notably, these groups often have less diverse gut microbiomes [79]. There is a well-established connection between the gut microbiome and the decline in health associated with aging [80]. As individuals grow older, they experience shifts in microbiome diversity and an increase in pro-inflammatory conditions. In older adults, the microbiome composition changes from being dominated by *Firmicutes*, which are common in younger individuals, to including genera such as *Alistipes* and *Parabacteroides* [81]. The gut microbiome in the elderly is characterized by considerable individual variability, especially in the presence of *Faecalibacterium*, *Ruminococcus*, and *Clostridium* clusters IV and XIVa. This diversity may help explain the varying effects of viral infections in older individuals [82]. Specific patterns of microbiome changes have been observed in patients with asthma and diabetes. Interestingly, asthma is less common among the comorbidities of critically ill SARS-CoV-2 patients [82]. Studies have indicated that severe asthma management and sputum neutrophilia are associated with the phylum *Proteobacteria* [83]. In contrast, in chronic obstructive airway disease, there is a decrease in *Bacteroidetes*, including *Prevotella* [83]. Interestingly, H2-producing *Prevotellaceae* are found in high numbers in obese individuals who are at risk of developing type II diabetes [84]. Additionally, it has been shown that a high abundance of *Bifidobacterium*, known for butyrate production, in type II diabetes patients can improve glucose tolerance [85]. Notably, there are some intriguing, yet limited, findings regarding the prevalence of Prevotella in sequencing data from COVID-19 patients.

Understanding the influence of SARS-CoV-2 on the gastrointestinal tract requires the identification of the primary gut microbiota that interact with the virus. However, with 1500 different gut microbiota species, this task is highly complex. Pinpointing the specific species that affect SARS-CoV-2 pathogenesis remains a challenge without conducting human trials. The gut microbiome engages with SARS-CoV-2 through a range of direct and indirect mechanisms. These interactions may encompass processes such as genetic recombination, changes in virion stability, the enhancement or suppression of viral infections, cell attachment in a way that facilitates infection, and the inhibition of viral replication through immune regulatory mechanisms. For instance, type II interferon (IFN-γ) plays a crucial role in the antiviral response [86]. Microbial metabolic processes in the gut influence the production of cytokines. The microbiome can enhance chronic phase proteins and interferon signaling in lung cells to combat influenza infection. However, as seen in the case of SARS-CoV-2, the response of the body to infection can be excessive. In certain individuals, the reaction of the immune system to SARS-CoV-2 can trigger an excessive release of cytokines, leading to hyperinflammation, severe acute respiratory distress syndrome (SARDS), and failure of multiple organs. To date, a cytokine profile linked to the severity of SARS-CoV-2 disease has been identified, with elevated levels of interferon-γ-inducible proteins, along with various other cytokines [87,88,89,90,91,92,93,94,95,96,97,98,99]. Therefore, understanding the molecular pathways of host cytokines and microbiota components and bacterial responses to cytokine reactions could enable novel microbiome-based therapies for SARS-CoV-2 infections.

To investigate the connections between dietary components, microbiome impacts, susceptibility to infections, and severity of illnesses, a variety of methodologies must be utilized. This requires large-scale, well-powered international research. Such studies should include both COVID-19 patients and control groups, to collect clinical data, detailed dietary assessments, host genetic information, immune profiling, and multisite multitopic microbiome markers. From an international standpoint, these studies should cover populations from diverse regions, dietary practices, and environmental exposure. This extensive and collaborative approach is crucial for understanding the factors that influence the clinical outcomes of infections, and for developing targeted treatments and preventive strategies. Additionally, the potential moderating effects of high-fiber foods, freshly fermented foods, and diverse diets should be explored as preventive and mitigation strategies.

## 5. Nanotechnological Approach to Establish the Nexus Between SARS-CoV-2 Infection and Gut Dysbiosis

Before the Human Microbiome Project (HMP) began in 2007, research on the human microbiome was largely neglected. The primary aim of this initiative was to explore the core human microbiome and its connection to host physiological processes. Thanks to progress in sequencing and analytical methods, along with a 40% boost in non-HMP funding, a wide array of studies focusing on the microbiome have been carried out. Research from HMP and other investigations underscores the essential function of gut microbiota in sustaining overall health and its role in the onset of various diseases due to alterations in the composition of the gut microbiota. As a result, the human microbiome market is anticipated to grow from $380 million in 2022 to $6091 million by 2035, experiencing a compound annual growth rate (CAGR) of 23.8% throughout the forecast period of 2022 to 2035 [100]. At present, the only human microbiome treatments that have been approved are fecal microbiota transplantation (FMT) products. This technique involves the transfer of microbial communities from a donor’s feces to a recipient, which can be administered via oral capsules or rectal methods, such as enemas and colonoscopies. Ferring Pharmaceuticals’ Rebyota became the first FMT product to gain FDA approval for preventing recurrent Clostridioides difficile infection (CDI) in individuals aged 18 and older following antibiotic treatment. In November 2022, the FDA’s approval of human microbiome therapy marked a significant milestone for drug developers in the human microbiome market. In April 2023, the FDA approved a second FMT product, VOWST (SER-109), created by Seres and Nestle, for the treatment of recurrent CDI. VOWST is the first fecal microbiota product to receive FDA approval for oral administration. Although Rebyota and VOWST are intended for the same purpose, they are administered differently. Various companies are engaged in creating prescription drugs aimed at influencing the human microbiome to treat a broad spectrum of gastrointestinal and non-gastrointestinal ailments, with a particular emphasis on infectious diseases. Furthermore, several commercial test kits are available for the diagnosis and screening of diseases associated with the microbiome. Root Analysis delivers an in-depth report on the human microbiome manufacturing sector, detailing both in-house and contract manufacturing organizations and their facilities [100].

Nanoscience and nanotechnology offer significant advantages in microbiome research because the nanoscale size and tunable properties of nanomaterials and nanodevices allow direct interaction with the biological components of microbiomes at their operational scales. This alignment of scales creates opportunities for substantial advancement through the development of innovative nanoscale analytical tools. Progress has been made in developing model systems that support the creation of these tools and methods [101,102,103,104]. These systems can then be applied to more complex real-world scenarios. As nanoscience has evolved from atom imaging to the direct manipulation of structures and guided interactions, we now have the capability to control the materials, structures, and chemical functionalities across various scales [105,106,107]. While substrates and surface functionalization have traditionally been aimed at resisting bioadhesion, the intentional arrangement of chemical patterns can also facilitate the growth and patterning of systems, such as biofilms, in contact with nanoscale probes [108]. By integrating these strategies with tools from other disciplines, we can enhance our understanding of the microbiome. To effectively study the microbiome, it is crucial to miniaturize and parallelize essential tools, drawing on decades of advancements in nanotechnology, including nanofabrication, imaging systems, lab-on-a-chip systems, and the control of biological interfaces [109,110,111,112]. Smartphone cameras and other commercially available tools can be repurposed for this purpose. By guiding the advancement and parallelization of these tools, it is possible to access increasingly intricate microbiomes [113]. In recent years, imaging and sensing technologies have undergone a revival, presenting a range of powerful measurement methods. The Microbiome Initiative has become increasingly relevant in light of recent developments in analytical techniques. The progress in omics technologies, such as electron and optical microscopy, spectroscopy, cytometry, mass spectrometry, atomic force microscopy, and nuclear imaging, has enabled researchers to explore the intricate aspects of microbiome interactions, functions, and diversity of the microbiome. Multimodal smart systems can be developed by combining advanced imaging, spectroscopy, and sensing methods with big data analytics. These systems can meet the critical demands of microbiome research, such as precisely analyzing microbial interactions at significant spatial and temporal dimensions, identifying the diversity within microbial genomes, transcriptomes, proteomes, and metabolomes, managing microbiomes to explore their functions, evaluating the effects of interventions, leveraging their activities, and assisting in the identification of microbial dark matter, which refers to the 99% of microorganisms that cannot be cultured [114].

**Table 1:** Early detection of different VOC biomarkers induced by the SARS-CoV-2.

VOC Sample Source	Total Number of Patients (*n*)	Analytical Tools	COVID-19 Associated Biomarkers	Basal Level Change	Reference
Oral Breath	98	GC-IMS	Acetone, Isoprene, Heptanal, Propanol, Propanal, Butanone, Ethanal, Octanal	Increased	[115]
			Methanol	Decreased	
Expired air from endotracheal tube	28	PTR-MS	2,4-octadiene, Methylpent-2-enal, Nonanal, 1-chloroheptane	Increased	[116]
End-tidal breath	56	GC-IMS	Acetone Propanol	Decreased Increased	[117]
Direct Exhaled Breath	340	PTR-TOF-MS	NO, Butane, Acetaldehyde, Heptanal, Ethanol, Methanol, Propionic acid	Increased	[118]
Direct Exhaled Breath	26	GC-TOF-MS	Octanal, Nonanal, Heptanal, Dodecane, Tridecane, 2-pentyl furan	Increased	[119]

### 5.1. Sensing Platforms for the Detection of VOCs in Pathological Conditions

Nanotechnology has the potential to improve diagnostic tools, monitoring systems, and therapeutic strategies for COVID-19, particularly in the context of gut modulation by SARS-CoV-2. For instance, human breath contains volatile organic compounds (VOCs) that play a role in central metabolic processes. Electronic nose (e-nose) devices use sensor arrays to detect VOCs in breath samples, with each sensor exhibiting varying sensitivities. Although these devices do not identify specific VOCs, they provide an overall fingerprinting response. Although breath samples contain numerous VOCs, only a few have been identified as COVID-19 specific biomarkers. For a VOC to qualify as a COVID-19 biomarker, it must be consistently correlated with SARS-CoV-2 infection, as confirmed by multiple studies (Table 1). Since ancient times, physicians have employed various methods, including exhaled VOCs, to diagnose diseases (approximately 400 BC) [120,121]. This approach relies on VOCs with low molecular weights, which change in response to pathophysiological processes that alter metabolism [121,122,123,124,125,126,127]. VOCs can be identified in body fluids, including the headspace of impacted cells, blood, exhaled air, and other bodily fluids [122,128]. Exhaled breath is the most accessible method for monitoring health disorders [121,128,129]. It is non-invasive, promotes high compliance, has a low-complexity matrix, and its sampling can be safely repeated. Disease detection using exhaled breath has been demonstrated in infectiology [130,131,132], respiratory medicine [133,134,135,136,137,138,139,140], and oncology [141,142,143,144,145,146]. However, advancing exhaled breath analysis requires expansion of current analytical approaches for disease diagnosis and classification. Diagnosis involves recognizing the disease, and classification is crucial for understanding its etiology, pathogenesis, and therapy. The demand for innovative diagnostic technologies to address clinical challenges is increasing each year. Nanotechnology-based sensor arrays could bridge the gap between fundamental research and modern point-of-care practices [147,148,149], offering devices that are smaller, more user-friendly, and cheaper than others. However, this approach is limited in detecting specific VOCs amid interfering gases, necessitating the time-consuming development of highly selective receptors [150]. Although advances have been made in VOC detection using selective nanomaterial-based methods, these methods are applicable only to a narrow range of diseases. Most diseases cannot be identified by individual VOCs alone, and the synthesis of nanomaterials that are selective for each VOC remains challenging, particularly for nonpolar compounds. VOCs sensors can probe various analyte-sensor interactions, generating different molecular specificities and responses [150]. While some interactions exhibit cross-reactivity and are less specific, others are tailored to a limited number of chemicals. These variations have resulted in the development of two sensing approaches: selective and cross-reactive.

#### 5.1.1. Selective Sensing

This method prioritizes the identification of specific VOCs among interfering gases by utilizing precisely engineered and highly selective receptors [151]. This approach is advantageous because it facilitates confirmed detection while somewhat diminishing the impact of interfering signals. To date, most selective gas sensors documented in the literature have focused on detecting reactive gases (e.g., nitric oxide and hydrogen peroxide) and certain VOCs (e.g., acetone) [152,153,154,155]. Nonetheless, identifying less reactive VOCs remains challenging, primarily because of the physicochemical similarities between VOCs within complex mixtures. Therefore, to achieve greater selectivity for the targeted VOCs, it is essential to establish strong probe-analyte interactions, such as those formed through coordination or covalent bonds, which offer more specificity than interactions such as van der Waals or Dipole–Dipole forces. Nonetheless, this approach may compromise the reversibility and recovery of the sensors because of their strong binding. Other methods to improve selectivity include Metal–Organic frameworks, which possess a 3D structure that enables specific interactions with VOCs while minimizing the effects of other gases [156]. As the requirements for sensing technologies evolve, it is imperative to redefine the selectivity of VOCs sensors to incorporate a more extensive array of related signals, including environmental factors such as temperature and pressure [157]. Undertaking this project is a challenging task that often necessitates the integration of various sensors and sophisticated data analysis methods. It is crucial to define the selectivity of a particular subgroup of chemicals or differentiate between various chemical types. Higher selectivity for the subgroup is more difficult because it is simpler to distinguish between chemical groups (such as aldehydes, acids, and amines) than to differentiate VOCs within the same chemical family (for example, amines with different aliphatic chains) [158,159]. Consequently, selectivity should be described in relative rather than absolute terms and provide clear information related to the size and the composition of the tested sample (i.e., a mixture of a few VOCs compared to breath containing thousands of VOCs).

#### 5.1.2. Cross-Reactive Sensing

**Figure 4:**
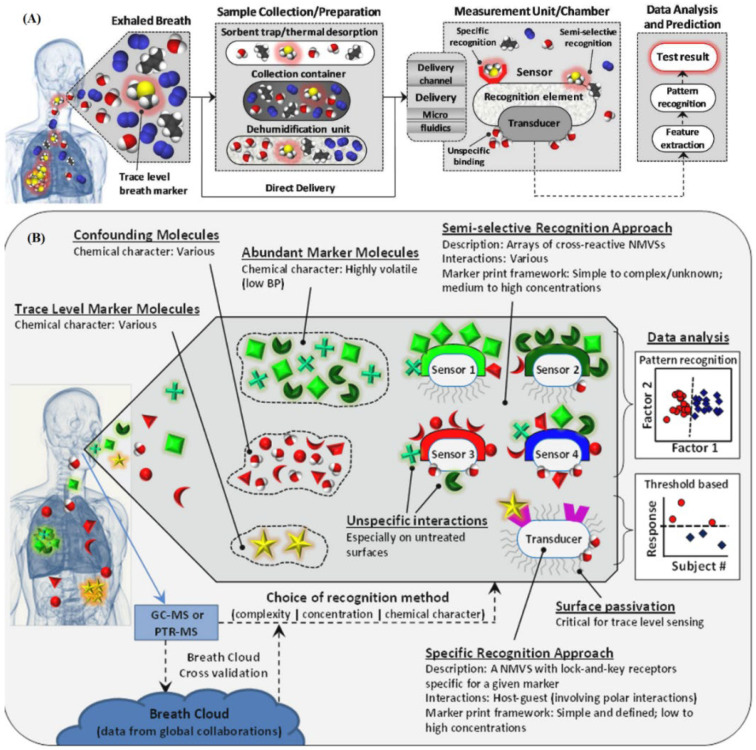
(**A**) The diagram illustrates the relationships between various frameworks of breath marker-prints and their associated sensing techniques, highlighting the distinction between specific and cross-reactive approaches. (**B**) Breath marker-prints, which are unique patterns of VOCs present in exhaled breath, can be analyzed using different sensing methods depending on the desired outcomes and characteristics of the biomarkers being investigated. Reproduced with permission from ref. [166].

In the analysis of intricate mixtures of VOCs, such as those present in breath and outdoor air, cross-reactive sensing approaches are often favored over the use of selective sensors. This alternative method mimics the olfactory system, which is responsible for our ability to smell, by employing arrays of sensors that are broadly cross-reactive in combination with artificial intelligence (AI) [160,161]. Termed the “electronic nose” or “artificial nose,” this method employs a sensor array capable of detecting all or a significant subset of VOCs within the targeted compound mixture [162]. Although these sensors are sufficiently diverse to yield unique responses to any given VOC in a mixture, strict selectivity is not necessary. By synchronizing the responses from various sensors, analyte-specific response patterns or “fingerprints,” can be discerned using classification and pattern recognition algorithms [163]. This approach offers numerous advantages, including high detection limits, broad dynamic ranges, and minimal sensitivity to variations in chemical and physical backgrounds. Artificial nose sensors based on cross-reactive approaches have been successfully applied in disease diagnosis and food monitoring [164,165]. Although the current focus on precise odor and molecular recognition is shifting towards artificial nose systems, the selectivity and specificity for particular VOCs are still highly valued. Consequently, an optimal sensor array should include a diverse set of sensor elements that possess high specificity for the intended analytes and exhibit a broad spectrum of chemical interactions (Figure 4) [166].

#### 5.1.3. Application of Nanomaterials for VOC Sensor Fabrication

**Figure 5:**
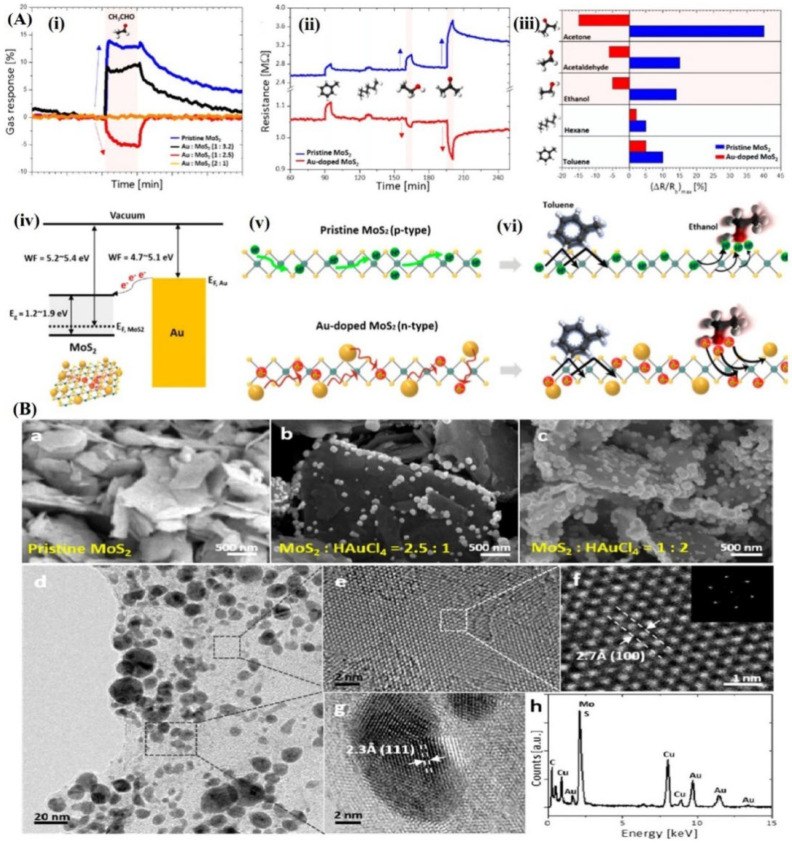
The tunable properties of MoS_2_ for VOC detection are affected by the introduction of gold doping. (**A**). (**i**) The variability in resistance in gold-doped MoS_2_ sensor upon exposure of acetaldehyde. (**ii**) resistance variation, and (**iii**) normalized response of pure MoS_2_ and gold-doped MoS_2_ sensors upon exposure to volatile compounds. (**iv**) Electronic band representing, MoS_2_ accepting electron from AuNPs which showing n-doping. (**v**,**vi**) Schematic illustrating the optimization of VOC detection through Au-induced n-doping. (**B**). (**a**–**c**) Scanning Electron Micrographs of the MoS_2_ and gold-doped MoS_2_ films synthesized at various HAuCl_4_ concentrations (MoS_2_/HAuCl_4_—ratios of 2.5:1 and 1:2). (**d**) Transmission Electron Micrographs showing the AuNPs adsorbed MoS_2_ film. (**e**,**f**) High Resolution Transmission Electron Micrograph of the gold-doped MoS_2_ film, revealing hexagonal lattice symmetry. (**g**) AuNPs on the MoS_2_ layers, demonstrating corresponding to the (111) plane of gold. (**h**) EDX spectrum of Au-doped MoS_2_. Adopted with permission from ref. [178].

Nanomaterials, characterized by dimensions ranging from 1 to 100 nm, can manifest in a variety of shapes, including rods, horns, spheres, tubes, particles, and fibers [167,168,169]. These materials are pivotal in the field of nanotechnology because they allow for the investigation, characterization, and analysis of materials with diverse geometries for numerous applications [167]. These materials can be utilized independently or as composites in sensing applications [170,171]. Owing to their ‘nano’ size, nanomaterials demonstrate low cytotoxicity, quantum effects, and a high surface to volume ratio. These attributes result in surface atoms with features such as superior molecular adsorption, enhanced biochemical activities, high catalytic activity and electrical conductivity [171]. Several techniques, such as FTIR, GC-MS, non-dispersive infrared spectroscopy, surface acoustic wave, chemiluminescence, along with electrochemical, colorimetric, and selected ion flow tube (SIFT) methods, have been applied to detect VOC biomarkers [172]. Although these methods are quick to detect, they encounter issues such as the size and expense of the equipment, the requirement for trained staff, and lengthy procedures [173]. These issues can be alleviated by employing real-time, cost-effective nanomaterial-based sensors for exhaled breath analysis [174]. Nanomaterials for VOC sensing are categorized as zero-dimensional, one-dimensional, two-dimensional, and 3D nanoarchitectures [175]. These hybrid systems integrate nanometric components that combine organic and inorganic elements, and portray properties that are challenging to achieve with conventional materials [176,177]. The integration results in characteristics shaped by factors such as size, composition, and form. The gas-sensing ability of MoS_2_ was enhanced by functionalizing it with AuNPs, which was achieved by decorating chemically exfoliated MoS_2_. The n-doping effect facilitated electron charge transfer from Au–MoS_2_, enabling the adjustment of MoS_2_ for detecting hydrocarbon-based VOCs. This innovation addresses the limitations of previously fabricated MoS_2_-based sensors, which typically exhibited single response to VOC analytes (Figure 5) [178]. Xiang et al. developed sensors using MWCNT fabricated with metal oxide nanocrystals via atomic layer deposition [179]. Hall Effect measurements indicated that the MWCNTs exhibited p-type behavior, confirming the formation of p-n junctions with the n-type ZnO nanocrystals. The electron-donating properties of ZnO result in a strong response to toluene at room temperature, highlighting its selectivity for volatile organic compound gases [179]. Additionally, two-dimensional molybdenum ditelluride (MoTe_2_) has demonstrated sensitivity for detecting ketone compounds [180]. The MoTe_2_ field-effect transistor exhibited unique sensing behavior towards ketone compounds, showing different responses before and after UV light activation, unlike its reaction with other VOCs. This feature facilitates the selective identification of ketone molecules in gas mixtures. The activation by UV light enhanced the sensitivity and lowered the detection limit for acetone to approximately 0.2 ppm. Additionally, the MoTe_2_ FET demonstrated a consistent sensing performance even in environments with high humidity [180].

**Figure 6:**
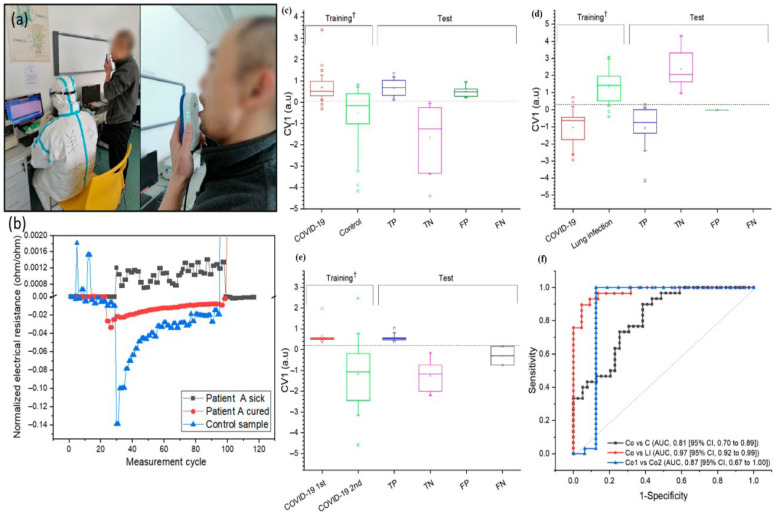
(**a**) Depiction of breath sample collected from COVID-19 patients in Wuhan, China. using a portable breath analyzer. (**b**) Typical response pattern of sensor 7 for three different breath samples. The normalized electrical resistance plotted against measurement cycle with units representing one cycle. Samples from infected individuals showed a positive change, whereas those from the recovered and control groups exhibited negative charges. The diagnosis of COVID-19 was determined by analyzing the cumulative responses of the breath samples. Panels (**c**–**e**) present the classification of data based on cumulative sensor responses, illustrated by the canonical variable from the discriminant analysis. (**e**,**f**) Box plots show the first canonical score for both the training set and the test set. ^†^ *p* < 0.0001. Adopted with permission from ref. [184].

Researchers have developed a method for fabricating biodegradable paper sensors coated with MoS_2_ and functionalized with nanoparticles such as Au, Pd, and Pt [181]. These sensors are designed to selectively detect low concentrations of VOCs. The MoS_2_ layer was grown on cellulose paper through a two-step hydrothermal process, and noble metal nanoparticles were applied via spray coating [181]. The sensors were tested for seven VOCs, including ketones, alcohols, and aromatic hydrocarbons, at 100 ppm. The Au–MoS_2_ and Pd–MoS_2_ sensors exhibited selectivity of 50.5% and 45.7% for acetone and benzene, respectively. They exhibit stable baseline resistance, low sensitivity to humidity (<75% RH), and response and recovery times of 3–6 min at 50 °C [181]. In another approach, researchers employed carbon nanotubes with polypyrrole-modified platinum electrodes to immobilize copper and zinc superoxide dismutase to detect nitric oxide in exhaled breath and H_2_O_2_-stimulated endothelial cells [182]. Gouma and Kalyanasundaram introduced a nanostructured probe of monoclinic tungsten trioxide (WO_3_) to detect low concentrations of NO amid interfering VOCs such as ethanol, methanol, isoprene, acetone, and CO [183]. The WO_3_ probe can detect NO concentrations of 1, 300, and 500 ppb, which are comparable to the levels found in human breath, although lower NO concentrations typically indicate various diseases. The sensitivity of the WO_3_ probe to NO originates from its chemical affinity for oxidizing molecules in a gas mixture [183]. A novel approach for detecting COVID-19 infection involves a breath-based device with nanomaterial-based hybrid sensors to identify disease-specific biomarkers [184]. A clinical study conducted at China, in March 2020 included 49 COVID-19 patients, 58 healthy patients (controls), and 33 non-COVID-19 lung infection patients (controls). The results demonstrated that nanomaterial-based sensors could differentiate between groups with high accuracy, achieving 94% accuracy in distinguishing patients from controls, and 90% accuracy between COVID-19 and other lung infections. Additional validation is required; however, this technology could reduce unnecessary confirmatory tests, and alleviate the hospital burden by providing screening at point-of-care facilities (Figure 6) (Table 2) [184].

### 5.2. Nanobiosensors for Gut Microbiota-Related Metabolites

The GI tract hosts a diverse array of culturable and un-culturable microorganism, such as archaea, bacteria, and some species of eukaryotes, known as the “gut microbiota”, which have co-evolved with humans to establish complex and mutually advantageous relationships [201]. The GI tract hosts over 10^14^ microorganisms, outnumbering human cells tenfold, and surpassing the genetic content of the human genome ~100-fold. There has been a recent surge in interest regarding intestinal flora, owing to its association with neuro-developmental disorders and various human diseases, such as IBS, IBD, metabolic complications such as obesity and diabetes, and luminal disorders, including allergic reactions. Gut microbiota is essential for maintaining an individual’s overall health [202]. It breaks down non-digestible materials such as fiber and intestinal mucus, encouraging the growth of gut microbiota that produce SCFAs and gases. Advanced diagnostic technologies can be used to identify microbial colonization within the human body by detecting microbial nucleic acid, protein, and antibody levels against specific antigens. Techniques such as ELISA [203], PCR [204], and FISH [205] have been used to examine the human gastrointestinal microflora. Microarray techniques, including DNA [206], oligonucleotides [207], and phylogenetic microarrays [208], allow for the simultaneous detection and quantification of thousands of genes, and target sequences, in a reduced timeframe. Although traditional analytical instruments are useful, they encounter obstacles such as being expensive, not easily portable, requiring skilled operators, and involving lengthy processes. On the other hand, miniaturized and portable diagnostic tools, like biosensors, are becoming popular in healthcare systems [209]. Compared with conventional bioanalytical tools, biosensors are more reliable, user-friendly, affordable and provide real-time PoC or PoN level health monitoring.

**Figure 7:**
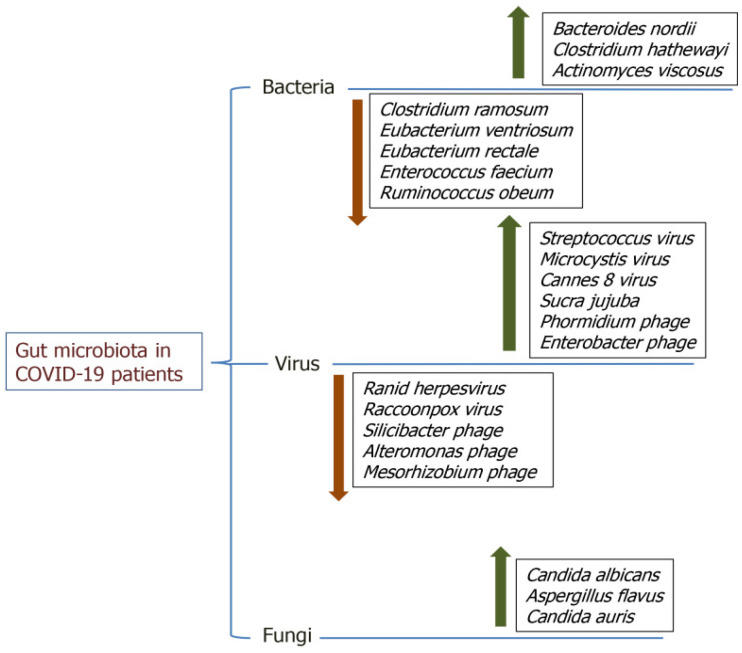
The illustration shows alterations in the gut microbiota of COVID-19 patients, emphasizing the differences in bacterial, viral, and fungal populations. Upward arrow indicating enrichment while downward showing reduction. Adopted with permission ref. [215].

The microbiome theranostics market is projected to expand from $506 million in 2022 to $899 million by 2025, indicating a CAGR of 22.1% [210]. This growth reflects increasing acceptance of products based on the microbiome. The market is categorized into two segments, therapeutics and diagnostics, both of which are anticipated significant growth. Notably, diagnostics is expected to witness the most significant rise, driven by the development of microbiome-related biomarkers in the field of oncology. Although microbiome-related tests are commercially available, they are not recommended as standalone diagnostics. Tools such as uBiome assess microbiome alpha diversity at various stages, facilitating the monitoring of compositional changes over time and their correlation with life events. Healthcare professionals can use these results for diagnostic purposes. Evivo provides a simple point-of-care test designed to detect *Bifidobacterium infantis* in new-born. Newborns acquire their gut microbiome during passage through the birth canal during delivery, which introduces *Bifidobacterium*. This introduction helps shield them from harmful bacteria and forms the foundation of the microbiome. The In Vivo test identified shifts in fecal pH when this bacterium colonizes the gut, offering quick results for infants with low levels of *Bifidobacterium*. The MinIon, a portable RT nucleic acid sequencer, is another promising diagnostic tool. Although not explicitly crafted for microbiome analysis, this device is capable of processing gut samples and taxonomic classification of the extracted microbiota by interpreting electrical signals from nucleotides as they traverse nanopores. Furthermore, it facilitates in situ sequencing, de novo sequencing, metagenomics studies, and epigenetic investigations; however, it is specifically designed for use by trained professionals. Various global initiatives have been initiated to investigate the human microbiome and its impact on health. In 2007, the Human Microbiome Project, financed by the NIH, utilized metagenomics to examine the commensal microbiota in healthy individuals across five anatomical locations: the nasal cavity, oral cavity, skin, gastrointestinal tract, and urogenital tract [211]. Although, a standard healthy microbiome could not be defined, NIH scientists are now investigating influence and possible linkage to the host in three clinical conditions: inflammatory bowel disease, pregnancy, and diabetes [212]. Similarly, the MetaHIT project, which completed in 2012, investigated the intestinal microbiota of healthy volunteers, categorizing them into three groups, or enteromes, based on the predominant gut-microbiota [213]. Another forward-looking initiative, American Gut Project (AGP), an open-source platform established under the umbrella of Human Food Project and the Earth Microbiome Project, accepts gut-biota samples deposition from individuals across world [214]. Their objective, aimed to investigate and establish a healthy baseline microbiome standard based on taxonomic diversity and functional diversity of the microbiome and its variation across human populations. Likely, several international initiatives have adopted a multi-omics approach to access the microbiome complexity and their interaction with host [214]. For instance, the open-source AGP project utilizes metagenomics and metabolomics to link participant samples with clinical (e.g., age) and lifestyle factors (e.g., dietary habits, smoking, or alcohol consumption). Notably, the occurrence of SARS-CoV-2 infection led to changes in the gut microbiota ecology of patients, in contrast to observations in the control groups. These alterations are affected by immune responses triggered during COVID-19 infection. Various studies have identified proliferation of atypical microorganisms, and a reduction in typical gut microbes, including bacterial, viral, and fungal populations, in individuals with COVID-19 (Figure 7) [215].

**Figure 8:**
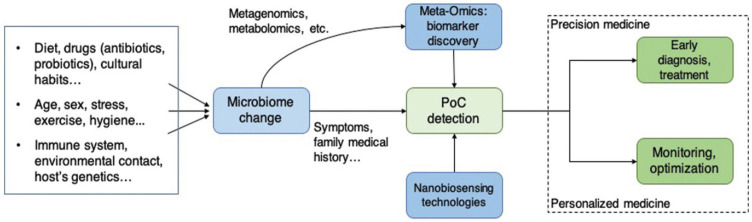
Nano biosensing was employed to detect microbiome-associated biomarkers, which were validated through multi-omics data. Devices utilizing nanobiosensors are promising for the prevention, early diagnosis, and monitoring of microbiome-related health issues. This is achieved by considering a range of clinical parameters, such as symptoms, antibiotic use, age, and family medical history, along with lifestyle factors such as diet, stress levels, and drug use. Adopted with permission ref. [217].

Moreover, the extensive data obtained by multi-omics approaches have facilitated the discovery of microbiome biomarkers linked to particular clinical conditions and diseases, thus providing new perspectives on diagnostic methods [216]. This progress paves the way for the development of rapid and sensitive sensors to monitor microbiome-related biomarkers, both in clinical settings and at home, potentially transforming the diagnosis and daily management of patients with various illnesses (Figure 8) [217]. Biosensors are rapidly becoming essential tools for point-of-care testing of both harmful and beneficial gut microbes, as well as gut microbial metabolites. Nonetheless, their advancement in the realm of the gut microbiota has not kept pace with other fields. This slower progress is attributed to several technical challenges, including (i) the current inability to culture most gastrointestinal microbes In Vitro, (ii) the complexities of isolating and performing standard assays on human samples, (iii) difficulties in accurately replicating the gastrointestinal microbial ecosystem in artificial models, and (iv) the influence of various external factors, such as diet, on GM.

#### 5.2.1. Nanomaterial-Based Biosensing of Gut Microbiota

**Figure 9:**
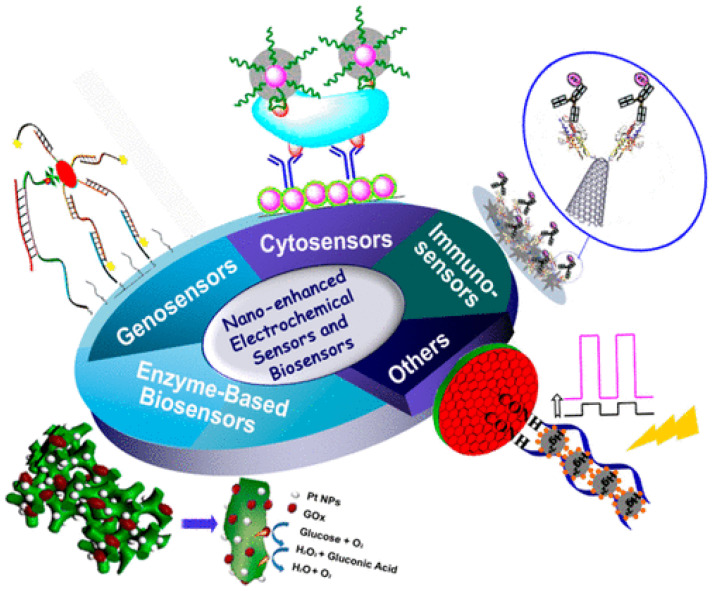
The schematic illustrates the use of nanomaterials and nanostructures in electrochemical sensors and biosensors, highlighting their applications in detecting small molecules, as well as in enzyme-based biosensors, Geno sensors, immunosensor, and cytosensors. Adopted with permission from ref. [218].

As already said, studies have shown that alterations in gut bacterial populations may be linked to underlying various diseases. To ensure treatment effectiveness, it is essential to regularly evaluate gut microbiota. However, traditional monitoring methods, such as NGS (Next-Generation Sequencing), are expensive and time-intensive. Consequently, advances in nanotechnology and nanoscience have demonstrated that the use of nanomaterials for electrochemical signal enhancement can improve the sensitivity and selectivity of electrochemical sensors and biosensors (Figure 9) [218]. The choice of electrode material is pivotal in the development of highly selective electrochemical sensing platforms designed to detect target molecules using various analytical techniques. Additionally, the integration of functional nanomaterials enhances signal transduction by leveraging their catalytic activity, electrical conductivity, and biocompatibility to produce synergistic effects. They can amplify biorecognition events using signal tags, leading to the development of highly sensitive biosensors. Research on functional electrode materials and electrochemical methods has expanded the applications of electrochemical devices. Walcarius et al. emphasized recent advancements in engineered nanostructured materials for the strategic design of bio-functionalized electrodes and related biosensing systems [219]. Table 3 summarizes the different types of systems, their LOD, and structures of biosensors.

**Figure 10:**
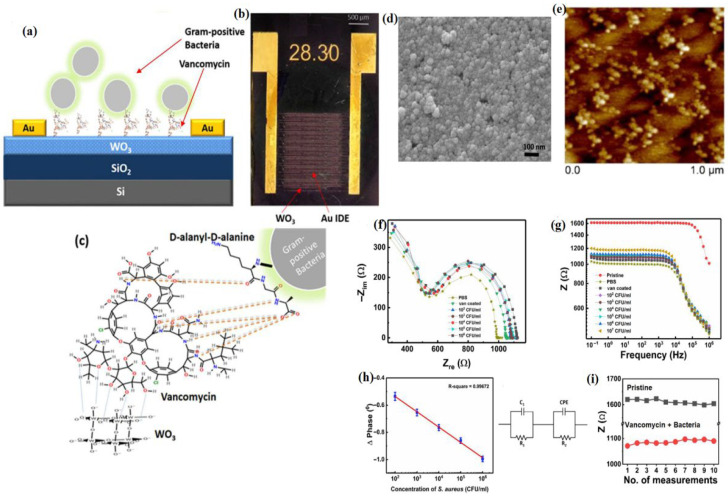
(**a**) Diagrammatic depiction of a vancomycin-coated WO_3_ IDE sensor illustrating the attachment of *S. aureus* to vancomycin on a WO_3_ IDE (cross-section view). (**b**) Visual image of the device, with 28.30 indicating the device number. (**c**) showing the interaction between the vancomycin attached on WO_3_ surface and D-alanyl-D-alanine sequence in the bacterial cell wall. (**d**) FESEM image of WO_3_. (**e**) Pristine WO_3_ with porous nanostructure. Impedance measurements for varying concentrations (CFU) of bacteria in PBS (pH 7.4, 10 mM) are shown in (**f**) Nyquist plot, (**g**) Bode plot, (**h**) phase change data, and (**i**) repeatability of the device over ten measurements. Adopted with permission from ref. [220].

In 2019, Singh et al. introduced an innovative impedance-based sensor capable of distinguishing between Gram (+) and Gram (−) bacteria, without any further labeling [220]. This sensor was engineered by embedding interdigitated gold electrodes in a slender tungsten oxide film. Following this, tungsten oxide (WO_3_) was modified with vancomycin, antibiotic that specifically binds to the peptidoglycan layer found in Gram-Positive bacteria. The vancomycin-enhanced tungsten oxide sensor exhibited a remarkable efficiency in capturing Gram-Positive bacteria. Impedance measurements effectively differentiated viable and non-viable Gram (+) bacteria. With a detection threshold of 102 colony-forming units (CFU) per milliliter, and a linear range spanning from 102 to 107 CFU/mL under physiological conditions, this vancomycin-coated sensor offers a rapid and sensitive label-free method for detecting Gram-Positive bacteria (Figure 10) [220].

The field of biosensing has seen significant advancements with the development of technologies like ingestible microbots, microfluidic-based sensors, and the Internet of Things (IoT). These innovations are notable for their robustness, compact design, multiplexing capabilities, programmability, and reliability, which enhance traditional biosensors and transform them into efficient POCT devices. Researchers are actively exploring responsive materials to further advance biosensor technologies [231]. Using these materials, ingestible capsules have been engineered to collect gut microbiome samples. The capsules are composed of a 3D-printed acrylic shell, hydrogel, and a PDMS membrane [232]. This non-invasive sampling method was validated using *E. coli*. Additionally, ingestible electronic capsules and self-powered biosensing systems have been designed to monitor various metabolites within the small intestine [233]. A novel ingestible probiotic biosensor was developed to diagnose gastrointestinal bleeding in swine, offering the potential to revolutionize gastrointestinal disease management [234]. The volatile molecules extracted from patient samples acted as biomarker. Further, a device known as an electronic nose, equipped with 13 electrochemical and optical sensors available on the market, was used to assess the microbial volatile metabolites present in urine samples from patients diagnosed with colorectal cancer [235].

#### 5.2.2. Nanomaterial-Based Biosensors for Gut Metabolites

Gut metabolites produced through bacterial metabolism serve as intermediates or end products. Key metabolites are aromatic amino acids and short-chain fatty acids, which originate from dietary fibers and fruits, and endogenous metabolites from bile acids and cholesterol. These metabolites interact with the autonomic nervous system via vagal and spinal nerves. They can enter the bloodstream and cross the blood–brain barrier, thereby influencing regulatory functions in the central nervous system [236]. The ANS (autonomic nervous system) plays a role in controlling gut permeability, motility, and immunomodulatory processes, which in turn affect bacterial composition and metabolites production. These metabolites are associated with neurodegenerative, neuroinflammatory, and neuropsychiatric disorders (e.g., Parkinson’s disease, Alzheimer’s disease, and autism spectrum disorders) [237,238].

**Figure 11:**
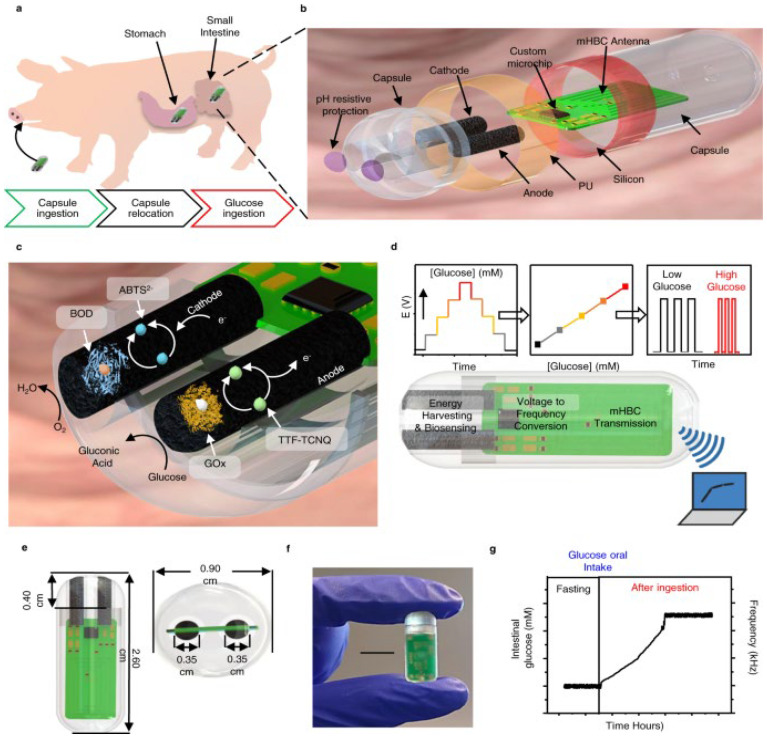
(**a**) Illustration of the capsule sensor within the pig model. (**b**) Capsule sensor. (**c**) Composition and chemical reactions of the BFC sensor. (**d**) Mechanism for signal conversion: Initially, glucose levels are converted into voltage, which is then changed into mHBC modulation frequency and transmitted wirelessly to an external receiver. (**e**) Capsule and BFC electrodes. (**f**) Image of the capsule with a 1 cm scale bar. (**g**) Results from in situ experiments conducted on a pig model after oral glucose consumption. Adopted with permission from ref. [233].

The interest in monitoring metabolites in clinical research is increasing. Owing to poor water solubility, metabolites exist in low concentrations in biological fluids, making accurate measurements difficult using current techniques. Several techniques, such as chromatography, Raman spectroscopy, SERS spectroscopy, and electrochemistry, have been established [239]. SERS technology identifies biological and chemical substances through vibrational patterns, advancing biomedical applications like POCT, précised imaging, molecular detection, and cancer diagnostics. However, noble metals and fixed probes limit SERS based detection at the molecular level [240,241]. HPLC has been widely used to detect metabolites. Sample preparation and extraction are critical in HPLC. Solid-phase extraction (SPE) and solid-phase micro extraction (SPME) are solvent-free pretreatment methods for HPLC. Selecting appropriate SPE and SPME sorbents is crucial because they affect the extraction efficiency and selectivity [242]. Despite their high sensitivity, these methods are complex, time-consuming, costly, and require sophisticated equipment and skilled personnel, making them impractical for routine measurements [243]. Wang Et Al. introduced an electrochemical sensing platform using a nanotip array for efficient measurement of indole derivatives [244]. The team created silicon nanotip arrays with controlled submicron gold-coated structures to fabricate nanotip electrodes. The electrochemical signal was amplified by incorporating AgNPs. Distinct oxidation peaks for indole, tryptamine, and indoxyl sulfate have been identified using DPV, enabling detection at nanomolar levels [244]. Three distinct 2D germanene-based, Ge–H, Ge-CH3, and Ge–C3-CN, nanomaterials were utilized as an impedimetric immunosensors to identify gut-derived metabolites linked to neurological disorders, such as kynurenic acid and quinolinic acid [245]. These immunosensors function through an indirect competitive process using disposable chips with printed electrodes. The primary antibody binding sites are occupied by both BSA-linked antigens on the electrode surface and free antigens present in the solution. Among these, Ge–H stands out for its exceptional bioanalytical performance in detecting KA and QA, achieving detection limits between 5.07 and 11.38 ng/mL (26.79 to 68.11 nM) and offering a faster reaction time compared to previous methods. The Ge–H competitive impedimetric immunosensor demonstrated slight cross-reactivity, and high reproducibility (RSD = 2.43–7.51%), and maintained stability for up to one month at 4 °C [245]. Lim et al. (2024) introduced a smartphone-based method for directly detecting metabolites in the gut, providing a faster and more cost-effective alternative to traditional methods [246]. This novel “turn off-to-on” fluorescence sensing technique, which used graphene quantum dots with copper (II) ions, not only offered sensitive detection with reduced analysis time but also effectively distinguished urolithin metabolites. Additionally, it demonstrated strong selectivity even in the presence of other reducing agents and phenolic analogs [246]. In a recent study, Paz et al. (2022) developed a novel ingestible biosensing system that functions without a battery and is specifically designed to monitor metabolites within the small intestine (Figure 11) [233]. To validate this system, the authors investigated variations in intestinal glucose levels using a porcine model. The system is driven by a self-powered glucose biofuel cell/biosensor incorporated into a circuit that supports biosensing, energy harvesting, and wireless data transmission tasks. This is achieved using a power-to-frequency conversion technique that leverages magnetic interactions with the human body [233].

SCFAs generated by intestinal bacteria enter the bloodstream and play crucial roles in human health. Although variations in SCFA levels have been linked to a range of diseases [247], their use in diagnostics is restricted because of the requirement for elaborate sample processing and expensive detection technologies. In 2023, Yavarinasab et al. developed an innovative electrochemical sensor that enables real-time quantitative SCFA measurements from complex liquid samples in less than two minutes, without the need for extraction, evaporation, or destruction [248]. This sensor employs an impedance-based mechanism, and is specifically designed to detect propionic acid, acetic acid, and butyric acid, which together make up over 85% of the SCFAs in the intestine. The sensor construction involved the deposition of ZnO and PVA onto a micro-fabricated interdigitated gold electrode, at physiologically relevant SCFA concentrations (0.5–20 mg/mL). Unlike, previous sensors that detect these acids only in gaseous form through evaporation, this sensor can detect them directly in the liquid phase at room temperature [248]. Using a similar approach, a different array of biosensors was engineered for the detection of L-lactate, employing flavocytochrome b2 (Fcb2) as the bio-recognition component and electroactive nanoparticles to immobilize the enzyme [249]. The enzyme was sourced from the thermophilic yeast *Ogataea polymorpha*. It was confirmed that the reduced form of Fcb2 directly conveys electrons to graphite electrodes. Additionally, the electrochemical interaction between the immobilized Fcb2 and the electrode surface was enhanced using redox nanomediators, which were both attached and mobile. These biosensors are notable for their high sensitivity, rapid response times, and low detection thresholds. A particularly effective biosensor, featuring co-immobilized Fcb2 and gold hexacyanoferrate, reached a sensitivity of 253 A·M^−1^·m^−2^ without the necessity for freely diffusing redox mediators and was used to assess the L-lactate content in yogurt samples [249].

## 6. Concluding Remarks

COVID-19 has been shown to affect the gastrointestinal system, leading to an imbalance in the gut microbiota. Such disruptions can have significant health consequences. SARS-CoV-2 can directly infect intestinal cells through ACE2 receptors, causing inflammation and altering the gut environment. These changes can decrease the number of beneficial bacteria, and increase that of harmful microorganisms. The effects of COVID-19-induced gut dysbiosis extend beyond the gastrointestinal symptoms. Research suggests that this imbalance may exacerbate COVID-19 symptoms and affect the long-term health outcomes. A healthy gut environment plays an important role in the functionality of the immune system, and its disruption can impair normal body functions. Additionally, gut dysbiosis is linked to systemic issues, including inflammation, metabolic disorders, and neurological symptoms, associated with prolonged COVID. The gut microbiome is a complex ecosystem that comprises thousands of bacterial species. Developing nanobiosensors to identify and differentiate microbial species and strains is challenging. The diversity of the gut microbiota complicates the creation of specific sensors to capture this environmental complexity. Translating biological interactions between nanobiosensors and the gut microbiota into measurable signals is complex, and requires advanced engineering techniques for reliable signal mechanisms and data interpretation. Developing sensors to obtain real-time information on microbial populations throughout the gastrointestinal tract remains a challenge. Creating nanobiosensors with high selectivity for specific microbes, while minimizing cross-reactivity, is complex because the compounds in the gut can interfere with sensor readings, necessitating careful design. Producing nanobiosensors on a reasonable scale and at cost is crucial; however, current fabrication techniques are cost-ineffective and difficult to scale up, limiting their commercial applications. Navigating regulations for new nanobiosensing technologies, and ensuring safety compliance, presents significant challenges, along with ethical concerns about data privacy and the potential long-term effects of nanomaterials on the body. Developing nanobiosensors compatible with existing diagnostic tools and medical practices is essential and requires compatibility with data management systems and user-friendly interfaces for healthcare professionals. Maintaining the accuracy of nanobiosensors over time is necessary, necessitating the development of in situ recalibration methods, or stable sensor designs, for continuous monitoring. These challenges underscore the complexity of the development of nanobiosensors for gut microbiota analysis. Addressing these issues requires collaboration among microbiologists, nanotechnologists, engineers, and medical professionals to create innovative solutions.

## Figures and Tables

**Table 2:** Some nanomaterials used for the VOC biomarkers sensing.

Nanomaterial	Size/Shape	VOCs	LOD (ppm)	*T_res._* (s)	Temperature	Reference
ZnO	Thin film	NO_2_	5–100	4.1	200 °C	[185]
ZnO	Flower-like microstructure	CH_3_CH_2_OH	50	8.3–12.43 s	RT	[186]
ZnFe_2_O_4_/ZnO	Flower-like microstructure	CH_3_COCH_3_	50	2	250 °C	[187]
ZnO/CuO on carbon substrate	Nano flower	NH_3_	5	4.1	RT	[188]
ZnO QDs	Quantum dots	Isoprene (2-methyl-1,3-butadiene)	1	8.0	150 °C	[189]
ZnO@CuO	Sphere/nanoparticle	H_2_S	10	33	RT	[190]
ZnO/Zn_2_SnO_4_	Micro flowers	CH_4_	400	10	250 °C	[191]
Co–doped ZnO	Nanofibers	CH_3_COCH_3_	100	4–6	360	[192]
Pd@ZnO	Nanosheets	CH_3_COCH_3_	1.9	30	340	[193]
Au/ZnO	Nano hybrid	CH_3_COCH_3_	1.7	15	270	[194]
NiO–decorated ZnO	Micro flowers	CH_3_COCH_3_	1.9	3.6	300	[195]
Au@ZnO	porous single-crystalline ZnO nanoplates	Isoprene	50	30	360 °C	[196]
Pd@TiO_2_	TiO_2_ nanorods	Isopropanol	500–2000	4.4	200	[197]
GO/SnO_2_	Nanofibers	HCHO	500 ppb	10	120	[198]
Au@NGQDs/TiO_2_	nanoporous/TiO_2_ nanospheres	HCHO	40 ppb	20	150	[199]
rGO NS SnO_2_ NF	SnO_2_ nanofibers with reduced graphene oxide (RGO) nanosheets	Acetone	100 ppb	≤1.3 min	350	[200]

**Table 3:** Different biosensor types, structures, and detection limit. (CFU) = colony forming unit; LSPR, Localized surface plasmon resonance; QD, quantum dot).

Nanomaterial	Target Biota	LOD	Reference
Vancomycin functionalized Tungsten oxide	*S. aureus*	80–100 cfu/mL	[220]
LSPR sensor	*Shigella* spp.	1.56 cfu/mL	[221]
QD based sensor	*E. coli*, *L. monocytogenes*, *S. Typhimurium*	102, 103, and 103 cfu/mL	[222]
colorimetric sensor	*Salmonella*	1 cfu/mL	[223]
Antibody-AuNPs	*Bifidobacterium bifidum*	2.1 × 10^2^ cfu/mL	[224]
OCMCS-Fe_3_O_4_ NPs	*C. jejuni*	103–107 cfu/mL	[225]
Bismuth-Fabricated carbon nanotubes	*H. pylori DNA*	0.72–7.92 μg/mL	[226]
Aptasensor	*S. aureus*	10 cfu/mL	[227]
Anti-*E. coli* antibody immobilized Gold Nanowire Arrays (GNWA)	*E. coli*	50 cfu/mL	[228]
DES/GO/AuNPs-FET	*E. coli*	3 cfu/mL	[229]
AuNPs and Magnetic nanoparticles	*Shigella* spp.	10^2^ cfu/mL	[230]

## Data Availability

Not applicable.
